# Unintentional Water Intake During Swimming and Post-Race Gastrointestinal Illness in Triathletes: Results from 6 Triathlons and 1294 Athletes

**DOI:** 10.3390/ijerph23030392

**Published:** 2026-03-19

**Authors:** Sander Bliekendaal, Miguel Dionisio Pires

**Affiliations:** 1 AERES University of Applied Sciences Almere, 1325 WB Almere, The Netherlands; m.dionisio@aeres.nl; 2 Deltares, Department of Freshwater Ecology and Water Quality, 2600 MH Delft, The Netherlands

**Keywords:** open water, swimming, health, triathletes

## Abstract

**Highlights:**

**Public health relevance—How does this work relate to a public health issue?**

- The study suggests that water intake during the swimming part of triathlons may be an exposure route for gastrointestinal illness, underscoring a recurrent environmental health risk in recreational water sports.

**Public health significance—Why is this work of significance to public health?**

- The observed relationship between the amount of water intake and post-race gastrointestinal illness may indicate limitations in current water quality monitoring practices and the need for improved athlete protection.

**Public health implications—What are the key implications or messages for practitioners, policy makers and/or researchers in public health?**

- The findings support the implementation of more frequent, near-race water quality monitoring and the development of athlete-focused preventive strategies to reduce unintentional water intake during open-water swimming.

**Abstract:**

This study aimed to investigate the relationship between water intake and post-race gastrointestinal illness in triathletes. Following a post-event survey approach, we evaluated the association between water intake and gastrointestinal illness in triathletes. We collected data among participants of six different triathlons in the Netherlands using an online questionnaire about personal characteristics (age, sex, swimming experience, chronic illness, and athletic level), the completed triathlon (length and duration), water intake, and illnesses during the 7 days following the triathlon. The associations between water intake and gastrointestinal illness were analyzed using generalized estimating equations logistic regression. In total, 1294 athletes participated in this study. The average rate of gastrointestinal illnesses per triathlon was 5.1%. In total, 75.3% of the athletes reported water intake during the race. The associations between water intake and gastrointestinal illnesses were significant. Triathletes with one to three sips of water intake reported 3.7 times more gastrointestinal illnesses (OR = 3.672, 95%CI: 1.316–10.242, *p* = 0.013) compared to those who did not ingest water. Triathletes with four or more sips of water intake reported 5.1 times more gastrointestinal illnesses (OR = 5.070, 95%CI: 1.740–14.767, *p* = 0.003). In conclusion, water intake was associated with an increased risk of post-race gastrointestinal illness. The results advocate for improved water quality monitoring and preventive measures in triathlon.

## 1. Introduction

Triathlon is a challenging multisport discipline that combines swimming, cycling, and running, and is experiencing an ongoing surge in popularity. Although the sport offers numerous health benefits, environmental exposures can lead to a range of post-race health issues [1]. For example, the swimming part of a triathlon is often conducted in open water bodies such as lakes, rivers, canals, or coastal areas. Athletes can then be exposed to waterborne pathogenic microorganisms, especially bacteria (e.g., *E. coli*) and viruses (e.g., norovirus) [2]. These exposures may cause gastrointestinal illnesses [3,4,5,6,7] through, for example, unintentional water ingestion [8].

Given that evidence suggests that water quality during triathlons plays a significant role in developing post-race illnesses, regulatory efforts are made to monitor water quality at the races’ locations [9,10,11]. Triathlon regulations state that for the event to take place, the water quality needs to be tested and graded as ‘good’ to ‘excellent’ in terms of pH, *E. coli* and enterococci concentrations, and presence of algal blooms [10,11]. However, because analyzation of samples in a lab is time-consuming, sampling is usually conducted approximately one week before the start of races [11]. Meanwhile, water quality can change rapidly, particularly in urban or agricultural areas where runoff and wastewater discharge are prevalent [8,12]. In those cases, athletes are then vulnerable to those environmental hazards at the time of the race.

Consequently, outbreaks of gastrointestinal illnesses in triathletes have been reported [13,14,15], and in one case, up to 42% of the athletes reported gastrointestinal illness symptoms within several days following the race [8]. Arguably, one of the most noteworthy examples of contaminated water during a triathlon race is the 2024 Olympic Games in Paris. The swimming part of the race, which was postponed once due to the high bacteria concentrations, took place in the River Seine. After the race, many athletes complained about the water quality, and several athletes were even hospitalized due to severe illness, referencing unintentional ingestion of polluted water [16].

However, only a few studies included water intake as a factor of developing post-race gastrointestinal illnesses among triathletes [8,13]. This underpins the need for a more comprehensive understanding of how water intake contributes to post-race illness in triathletes [17]. Hence, this study aimed to investigate the relationship between water intake and post-race gastrointestinal illness in triathletes. It was hypothesized that the incidence of post-race gastrointestinal illness was related to the amount of water intake during the race. Increasing insight into this relationship supports specifying water-related risk perception in triathlon regulating bodies, triathlon organizers, triathletes, and water authorities. Ultimately, this study aimed to contribute to the enhancement of athlete safety and support triathlon organizers to mitigate the impact of environmental hazards during open-water swimming.

## 2. Methods

### 2.1. Study Design

Following a post-event survey approach, we evaluated the association between water intake and gastrointestinal illness in triathletes after triathlon races. We collected data among participants of six different triathlons between May 2025 and September 2025 in four different cities in the Netherlands: Almere (2 triathlons), Amsterdam (2 triathlons), Ouderkerk aan de Amstel (1 triathlon), and Rotterdam (1 triathlon).

### 2.2. Respondents

The respondents in this research were all athletes participating in a triathlon. The respondents were informed about the goals and procedures of this study in an invitation letter sent by e-mail. Only athletes aged 18 and older were invited to participate in this study. Consent was obtained explicitly prior to participating in the study.

### 2.3. Questionnaire

The study’s invitation letter included a link to an online questionnaire to obtain data on their health status in the 7 days following the race. This was sent by the triathlons’ organization between 7 and 10 days after the race. The questionnaire remained open for responses for 14 days to limit recall bias. Following the International Olympic Committee’s consensus statements, we used the following definition for illness: *“Any complaint or disorder, not related to injury, including physical (e.g., flu), mental (e.g., depression), or social wellbeing”* [18].

After a short introduction, the questionnaire included questions about the personal characteristics age category (18–29 y, 30–39 y, 40–49 y, 50–59 y, ≥60 y), sex (male, female), experience in outdoor swimming (yes, no), chronic illness (yes, no), athletic level (beginner, advanced, elite), the length (meters) and duration (intervals of 15 min) of the swimming part of the triathlon, and water intake (no, yes, number of sips). It also included questions about illness during the 7 days after the event. In the case of illness, symptoms could be specified in a multiple-response format of 13 different symptoms, similar to previous studies [3,13,19]. See Table A1 for an overview of the questionnaire items. The symptoms nausea, vomiting, diarrhea, and stomachache were considered symptoms of gastrointestinal illness [6,18]. The other symptoms were also recorded but are not included in this study.

### 2.4. Statistics

The response rate was calculated by dividing the number of questionnaires received by the total number of invited respondents. The number of sips of water intake was categorized into one to three sips and more than three sips, which is in accordance with Joosten et al. (2017) [19].

The independent variable was water intake (no, yes, 1–3 sips, ≥4 sips) and the dependent variable was gastrointestinal illness (no, yes). The associations between water intake and gastrointestinal illness were analyzed in consecutive models, with no water intake as the reference. Model 1 consisted of a univariable analysis using generalized estimating equations logistic regression with correction of the analysis for clustering of the data within each triathlon. In model 2, we added the personal variables (i.e., sex, age category, experience in outdoor swimming, chronic illness, athletic level, and the duration of the swimming part of the race) into the model as covariables. To avoid overfitting of the model, we applied backward selection of the personal variables: variables with *p*-values higher than 0.2 were excluded, one by one, with the highest *p*-value first. This was repeated for all the levels of water intake. In all these analyses, an independent correlation matrix was used. Odds ratio (OR), *p*-values, and confidence intervals (CI) were used to assess the results’ significance. All statistical analyses were conducted using IBM SPSS version 29 (SPSS Inc., Chicago, IL, USA).

## 3. Results

**Figure 1:**
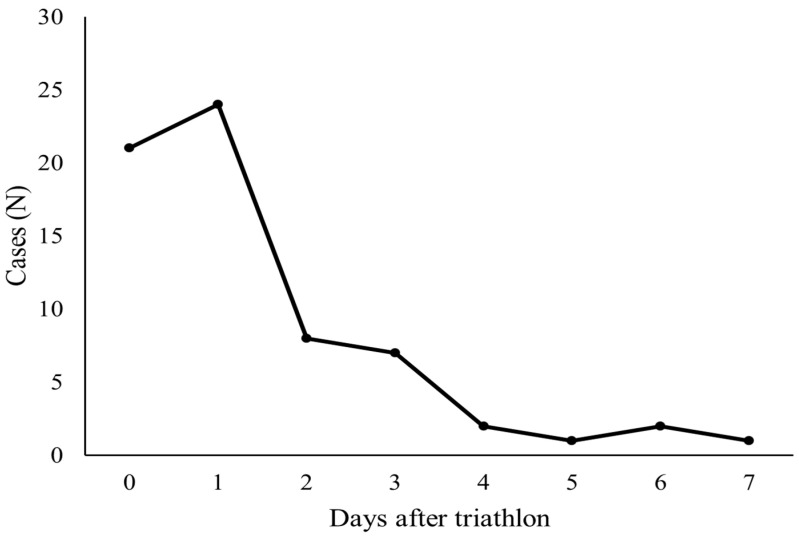
The onset of gastrointestinal illnesses.

In total, 1294 respondents were included in the study, which indicated an overall response rate of 18% (SD = 3.7) (Table 1). Respondent characteristics are presented in Table 2. The average rate of gastrointestinal illnesses was 5.1% (SD = 2.6), which varied from 0.5% to 7.6% between the triathlons (Table 1). The onset of gastrointestinal illnesses was most common on the day of the triathlon and the day after (Figure 1).

In total, 75.3% of the athletes reported water intake during the race, of which 32.2% ingested four or more sips (Table 2). The proportion of athletes who developed gastrointestinal illness without water intake, with 1–3 sips intake, and four sips or more intake was 1.3%, 4.8%, and 8.4%, respectively (Table 3).

Water intake was associated with the development of gastrointestinal illness, which was robust after model adjustment (Table 3). In the final model, any water intake was associated with a 4.36 times increased risk of gastrointestinal illness (OR = 4.355, 95% CI: 1.614–11.749, *p* = 0.004) compared to no water intake. The association between water intake and gastrointestinal illnesses was stronger in the group with four or more sips of water (OR = 5.070, 95% CI: 1.740–14.767, *p* = 0.003) compared to the group with one to three sips of water (OR = 3.672, 95% CI: 1.316–10.242, *p* = 0.013) (Table 3).

## 4. Discussion

In this study, we investigated the relationship between water intake during the swimming part of a triathlon and post-race gastrointestinal illness. We found a significant relationship between water intake and gastrointestinal illness. The intake of one to three sips of water was associated with a 3.7 times higher risk of developing gastrointestinal illness, and four or more sips of water intake was associated with a 5.1 times higher risk compared to no water intake. Our study is one of the few investigating this relationship in regular triathlons, as most of the literature on this subject involved beach users [20,21], open water or canal swimming events [3,14,19], or outbreaks in triathlons [8,13].

Our study indicated that post-race gastrointestinal illness is somewhat common in triathletes, as on average 5.1% of triathletes reported gastrointestinal illness within the week after the triathlon. This result fits within the range of findings of previous studies in triathlons and canal swimming events without water quality issues. In those studies, rates between 4% and 9% were found [3,6,8,19]. Compared to the results of studies in triathlons and canal swimming events where the athletes swam in polluted waters, our results are much lower than expected. In those studies, rates between 31% and 42% were reported [8,13,19].

Previous studies have also pointed out that water intake was associated with 1.3 to 3.1 times higher risks of gastrointestinal illness in triathletes and participants of canal swimming events [3,8,13,19]. In our study, even small amounts of water intake (i.e., 1–3 sips) were associated with a 3.8-times higher risk of gastrointestinal illness. This risk was even higher when four or more sips of water were ingested (i.e., 5.1 times). Thus, compared to the previous studies, the risks calculated in our study are relatively high. However, comparability to those studies may be limited because of their single-event analysis and, in some cases, issues with water quality [8,13] and a smaller sample size [13].

Despite those differences, the literature and this study’s results clearly demonstrated that the risk of gastrointestinal illness was related to the amount of water intake [13,19]. Thus, water intake during the swimming part of the triathlon may play an important role in the development of post-race gastrointestinal illness, even when the water quality was considered sufficient. This may be a common and overlooked problem in triathlons. It also raises the question of whether triathlon organizers and triathletes are aware of those risks and which preventive strategies may help mitigate them. Moreover, the results from this study reinforce the need to raise awareness on water quality and the monitoring of it to protect triathletes’ health during races.

### Strengths and Limitations

The strengths of this study were the multi-triathlon study design, a relatively short recall period, and a relatively large sample size. Also, as this study involved regular triathlons without water quality issues, the external validity of the findings was strengthened. Nevertheless, some limitations need to be considered when interpreting the results. First, using a questionnaire enabled us to conduct this study in a multi-event using few resources. However, this study is susceptible to bias because it relies on self-reported data. For example, athletes who became ill after the race may be more likely to respond to the invitation. The athletes could also have over- or underestimated their water intake, leading to misclassification. This could lead to type 1 errors. Also, data regarding the validity, reliability, and recall bias of self-reported water intake are absent in the literature, and the margin of error in this data is unknown. It is relevant to investigate this in future studies. Second, the response rates are relatively low. This could have led to overestimation of the results, given that epidemiological outcomes are related to the response rates [22]. Third, although our recall period was sufficient to capture gastrointestinal illnesses following triathlons, as gastrointestinal illness usually occurs within 3 days after exposure [23], it cannot be excluded that, in some cases, the gastrointestinal illness was caused by something else before or after the event. If this is the case, this may have caused underestimation of the relationship between water intake and gastrointestinal illnesses.

## 5. Conclusions and Implications

A significant relationship between water intake and post-race gastrointestinal illness was observed. Small amounts of water intake (i.e., 1–3 sips) were associated with a 3.7 times increased risk of developing gastrointestinal illness post-race. Higher amounts of water intake (four or more sips) were associated with a 5.1 times higher risk. Despite the relatively low gastrointestinal illness rates among triathletes in our study, these outcomes call for more awareness of the health risks associated with the intake of potentially contaminated water during the swimming part of triathlons. It also advocates for improved water quality monitoring and provides insights for the development of preventive measures in triathlon events. To protect the athletes’ health, intensified water quality monitoring up to the day of the triathlon is advised. Additionally, given that gastrointestinal illness is related to the amount of water intake, we advise involved parties (e.g., triathlon organizers, triathlon training centers, and triathlon coaches) to work on strategies to reduce water intake during open water swimming.

## Figures and Tables

**Table 1:** Number of participants and response rate per event.

Event (#)	Participants	Response		Illness Symptoms			
	N	N	%	Total		Gastrointestinal	
				N	%	N	%
1	728	157	21.6	12	7.6	5	3.2
2	1308	191	14.6	19	9.9	1	0.5
3	1032	205	19.9	34	16.6	13	6.3
4	920	114	12.4	9	7.9	6	5.3
5	2343	487	20.8	91	18.7	37	7.6
6	857	140	16.3	21	15.0	4	2.9
Total	7188	1294	18.0	186	14.4	66	5.1

N, Number.

**Table 2:** Respondent characteristics.

Variable	N	%
***Sex***		
Male	917	70.9
Female	377	29.1
***Age***		
18–29 y	220	17.0
30–39 y	327	25.3
40–49 y	379	29.3
50–59 y	269	20.8
≥60 y	99	7.7
***Outdoor swimming experience***		
No	202	15.6
Yes	1092	84.4
***Athletic level***		
Beginner	663	51.2
Advanced	606	46.8
Elite	25	1.9
***Chronic illness***		
No	1245	96.2
Yes	49	3.8
***Swimming distance***		
Short (400–1000 m)	340	26.3
Middle (1.500 m–1900 m)	761	58.8
Long (3800 m)	193	14.9
***Swimming time***		
Less than 30 min	490	37.9
Between 30 and 60 min	613	47.4
More than 60 min	191	14.8
***Water intake***		
No	320	24.7
Yes	974	75.3
1–3 sips	557	43.0
≥4 sips	417	32.2

N, Number; y, year.; m, meter.

**Table 3:** The associations between water intake and gastrointestinal illnesses.

Water Intake	Participants (N = 1294)			Model 1			Model 2		
	Total	Illness		OR	95% CI	*p*	OR	95% CI	*p*
	N	N	%						
No	320	4	1.3	Reference			Reference		
Yes	974	62	6.4	5.371	2.008–14.364	<0.001 *	4.355 ^1^	1.614–11.749	0.004 *
1–3 sips	557	27	4.8	4.025	1.508–10.740	0.005 *	3.672 ^2^	1.316–10.242	0.013 *
≥4 sips	417	35	8.4	7.238	2.581–20.301	<0.001 *	5.070 ^3^	1.740–14.767	0.003 *

N, number; OR; Odds Ratio; CI, Confidence Interval; *p*, *p*-value. ^1^ The variable chronic illness was removed from the model. ^2^ The variables chronic illness and experienced in outdoor swimming were removed from the model. ^3^ The variables chronic illness and athletic level were removed from the model. * Significant at *p* < 0.05.

## Data Availability

The raw data supporting the conclusions of this article will be made available by the authors on request.

## Appendix A

**Table A1:** The questionnaire items.

#	Question	Answer Options
1.1	In the period from [date 1] to [date 2] (the week after the [name triathlon], did you suffer from health complaints?	Yes; No
1.2	Did these health complaints arise in the period from [date 1] to [date 2] (the week after the end of the [name triathlon])?	Yes; No, I already had these complaints
1.3	Which health complaints have you experienced? (multiple options possible)	Nausea; Vomiting; Diarrhea; Abdominal pain; Fever (more than 38.5 degrees); Shivers; Headache; Muscle or joint pains; Eye complaints (red or sensitive eyes); Ear complaints or ear pain; Colds, coughs or shortness of breath; Itching or rash (red bumps); Other
1.4	How many days after the race did these complaints arise?	On the day of the race; After 1 day; After 2 days; After 3 days; After 4 days; After 4 days; After 6 days; After 7 days
1.5	How severe were these symptoms of illness?	Mild; Moderate; Severe
2	Did you participate in the swimming part?	Yes; No
2.1.1	What distance did you swim?	
2.1.2	How long did the swim part take you?	Less than 15 min; Between 15 and 30 min; Between 30 and 45 min; Between 45 min and 1 h; Between 1:00 and 1:15 h; Between 1:15 and 1:30 h; Between 1:30 and 1:45 h; Between 1:45 and 2:00 h; More than 2:00 h.
2.2.1	Did you, accidentally, ingest water while swimming?	Yes; No
2.2.2	How many sips do you think you have swallowed?	1; 2; 3; 4; 5; 6; 7; 8; 9; 10 or more
2.3	Where you trained in outdoor swimming?	Yes; No
2.4	What do you consider your level as a triathlete?	Beginner/Recreational; Advanced; Elite
2.5	What is your sex?	Male; Female
2.6	What’s your age?	18–29 years; 30–39 years; 40–49 years; 50–59 years; 60+
2.7	Did you have a chronic illness in the past year?	Yes; No
